# eBrain: a Three Dimensional Simulation Tool to Study Drug Delivery in the Brain

**DOI:** 10.1038/s41598-019-42261-3

**Published:** 2019-04-16

**Authors:** Yaki Setty

**Affiliations:** Gateway Institute for Brain Research, 3321 College Avenue, Davie, 33314 Florida USA

## Abstract

Neurodegenerative disorders such as Alzheimer’s and Parkinson’s disease are severe disorders with acute symptoms that gradually progress. In the course of developing disease-modifying treatments for neurodegenerative disorders there is a need to develop novel strategies to increase efficacy of drugs and accelerate the development process. We developed a tool for simulating drug delivery in the brain by translating MRI data into an interactive 3D model. This tool, the eBrain, superimposes simulated drug diffusion and tissue uptake by inferring from the MRI data with a seamless display from any angle, magnification, or position. We discuss a representative implementation of eBrain that is inspired by clinical data in which insulin is intranasally administered to Alzheimer patients. Using extensive analysis of multiple eBrain simulations with varying parameters, we show the potential for eBrain to determine the optimal dosage to ensure drug delivery without overdosing the tissue. Specifically, we examined the efficacy of combined drug doses and potential compounds for tissue stimulation. Interestingly, our analysis uncovered that the drug efficacy is inferred from tissue intensity levels. Finally, we discuss the potential of eBrain and possible applications of eBrain to aid both inexperienced and experienced medical professionals as well as patients.

## Introduction

The neurodegenerative disorders such as Alzheimer’s and Parkinson’s disease are severe disorders with acute symptoms that gradually worsen over time^1^. Due to the complexity of brain tissue and its essential function, developing disease-modifying treatments for neurodegenerative disorders requires arduous study of dynamic progression of the disease state^2–5^. The bulk of collected data, however, are snapshots at specific time points or pathology of a postmortem state. Therefore, the mechanisms in a living organism underlying the degenerative process and the way treatment strategies control them, remain largely unknown. Consequently, drug development in neurodegenerative research is slow and treatment paradigms are normally developed over repetitive trial and error cycles. To advance the field, there is a need to develop novel strategies to increase efficacy of drugs and accelerate the development process.

Various approaches, technologies and systems are being developed to study the transportation of pharmaceutical compounds into the brain^6–8^. We use computational and mathematical approaches to simulate and study brain mechanisms and to test complex drug delivery systems *in-silico*. Specifically, we simulate an intranasal delivery system in which the drug is infused into the brain through the nasal passage^9^. Our work is inspired by an ongoing clinical trial for Alzheimer’s patients^10,11^, in which insulin is delivered intranasally into the patient’s brain to enhance brain function and memory recovery. Preliminary results have shown increased glucose uptake in the brain tissue parenchyma and enhanced brain neuronal activity^12,13^. Developing this finding into a treatment requires determining a dosage that safely enhances cognitive recovery. Although this is clinically feasible, the necessary steps involved would require extensive testing to determine the most effective drug compound and dosing. Any trial would also need to account for avoidance of overdose, patient reaction, and potential side effects^14,15^.

Using a 3D simulation tool, we studied how a drug is diffused and absorbed in the brain. This tool allowed us to examine the relationship between cell stimulation, drug dose, tissue density, and tissue permissibility. This strategy has the potential to accelerate drug development and reduce trial and error cycles. This work will pave the way to a professional tool of design and analyze clinical trials and patient-specific protocols.

eBrain, the tool we developed for this study, is an in-house 3D computer-based simulation built using 3D Graphics technologies that are often used for products in the gaming industry. eBrain integrates physiological data, medical images for processing measurement input, and experimental knowledge into clinically relevant output^16^. eBrain supports *in-silico* analysis of the system dynamics under various conditions. We show how eBrain helps to study intranasal drug delivery and to screen optimal treatments over multiple possible scenarios. Furthermore, it will assist in efforts to create more focused clinical trials with reduced cohort sizes, thereby potentially minimizing the risk to human lives. Notably, eBrain can be personalized to each patient by processing data specific to each individual. eBrain is built on previous simulation strategies that have successfully predicted experimental results in various biological systems, some of which gained support by subsequent experimental study^17–22^. Several reviews have highlighted the potential of simulation in aspects related to neuroscience, e.g., migration, and symptom profiles^23,24^.

In this manuscript, section one details the design, the experimentally derived algorithms, and the set-up requirements of the framework of eBrain. Section two describes examples of diffusion and tissue uptake simulations for intranasal delivery with a comparison to experimental results. Section three mathematically characterizes eBrain internal functions and details various possible scenarios of tissue stimulation following different routes of administration. Section four discusses how eBrain analysis can be used to predict optimal drug delivery. Finally, section five highlights future features and extensions for eBrain and possible applications of the software.

## Section 1: Algorithms, Design and Set Up Requirements

### Three dimensional virtual model from MRI data

To develop eBrain simulations, we used the C++ programming language aided by tools from Epic Games’ Unreal Engine (www.unrealengine.com) framework. The MRI data in the simulation was obtained from the FOX Foundation database (https://www.michaeljfox.org/). Specifically, we used the MRI of subject 10874, a 73 year old female Parkinson’s disease patient dated 9/5/2014 and downloaded from the Parkinson’s Progression Markers Initiative (PPMI) database (http://www.ppmi-info.org/). The virtual MRI model overlies a 3D grid containing approximately 500,000 grid voxels (matrix of 257 × 80 × 24).

**Figure 1:**
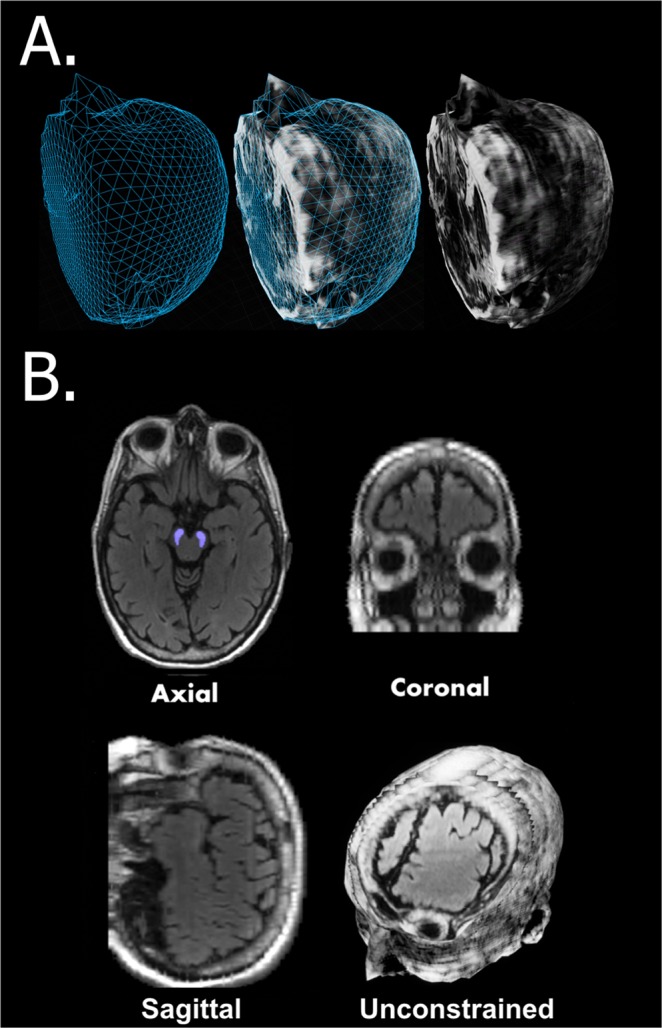
3D virtual model of Brain MRI. (**A**) The surface mesh generated by the MRI next to the rendered model. (**B**) Axial, Coronal and Sagittal plane as well as an unconstrained view of a ~45 degrees plane from a forehead. The substantia nigra area of the tegmentum is designated in blue. See also Supplementary Clip 1.

The 3D surface tightly encloses all points in 3D space that are contained within our volumetric material, then uses the volumetric material to render the MRI at all points (Fig. 1A). For internal views of the MRI, we created a ‘virtual screen’, a 3D plane located in front of the virtual camera and moving simultaneously with it. The volume material is processed in real time, therefore additional data can be layered on top of the MRI. The results are a completely unconstrained view of the MRI data, viewable from any angle, magnification, and from any position. Areas of interest (e.g.,nose, substantia nigra) are superimposed. This design allows us to dynamically select multiple layers of 3D data for comprehensive visualization. eBrain 3D visualization permits multiple perspectives including the traditional axial, coronal, and sagittal planes^25,26^. The camera moves seamlessly to slice the image over unconstrained views (Fig. 1B and Supplementary Clip 1). Each plane is projected into the three traditional planes, showing the three slices that correspond to the plane that is under inspection. eBrain thus allows tracking of tissues that are not aligned with any specific plane and are branched tissues from the stem area.

### Simulating drug diffusion in the brain via the intranasal delivery system

To simulate intranasal drug delivery in eBrain, we marked the delivery related locations within the nasal cavity on the MRI (specifically, the olfactory bulb and trigeminal pathways involving perineural and perivascular channels). The superimposed grid cells are set as the origin for calculating the diffusion of the drug quantity to be delivered. The current version simulates a passive transport of the drug; however, the simulation can be extended to support active transport and efflux transporter systems. This can be achieved by labelling the nasal epithelium in the MRI and formulating a mathematical equation to mimic the dynamics of the relevant transport system.

**Figure 2:**
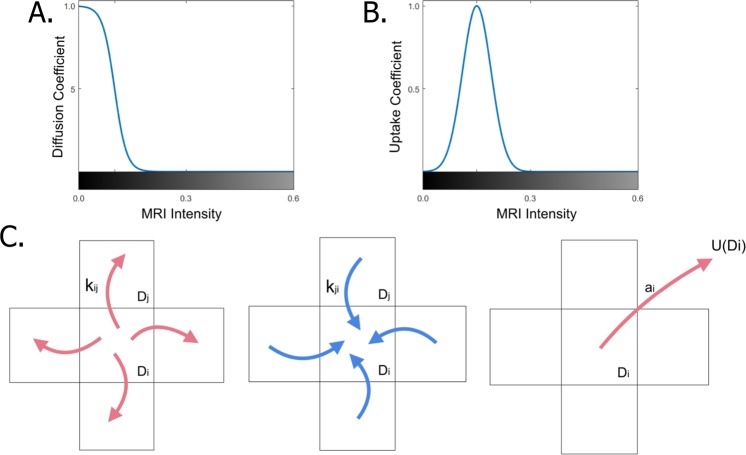
Algorithms design of the simulation. (**A**) Solution for the diffusion equation of a factor as function of MRI intensity from black to white. (**B**) Solution for the tissue uptake equation as function of MRI intensity from black to white. (**C**) Illustration of drug diffusion and absorption in the grid overlies the MRI.

For diffusion simulation of intranasal drug delivery, we assumed that the diffusion of the drug through the brain is driven by tissue density. Accordingly, in regions with the highest density where the MRI intensity is lightest (e.g., bones) we assumed diffusion is minimal. In regions with the lowest density, where the MRI intensity is darkest (e.g., liquid areas, CSF) we assumed diffusion is maximal. For grey areas, we assume that the diffusion coefficient varies with shade from minimal to maximal values. We designed the diffusion in the simulation by approximating the diffusion coefficient as a function of MRI intensity in each grid cell according to a reverse logistic function with midpoint = 0.1 and steepness = 60 (Fig. 2A). This assumption is supported by several experimental studies testing insulin diffusion and glucose uptake in brain tissue and hydrogels^27^. Obviously, in real-life brain tissue, other factors are involved in the diffusion process (e.g., the blood-brain-barrier and the vascular network). However, for the purpose of the intranasal delivery study, we omitted them. Future extensions of eBrain would cover these aspects creating a more comprehensive platform.

We used discretization of a master equation to simulate drug diffusion between the *i-th* voxel and the flux from the set of the adjacent voxels (Eq. 1). Each time-step, the simulation sums the flux in of the drug from the adjacent voxels and subtracts the flux from the *i-th* voxel to the adjacent voxels. The diffusion rate between two adjacent cells is determined based on their MRI intensity color.

1 $${D}_{i}^{n+1}={D}_{i}^{n}+{\rm{\Delta }}t\sum _{j\in N}{k}_{ji}{D}_{j}^{n}-{\rm{\Delta }}t\sum _{j\in N}{k}_{ij}{D}_{i}^{n}-{U}_{i}^{n}({D}_{i})$$

with ${D}_{i}^{n}$ being the drug concentration of the *i*-th voxel at the *n*-th iteration step, *j* being the index of an adjacent voxel, *N* being the set of neighboring voxels, ${k}_{ij}$ being the diffusion rate from the *i*-th voxel to the *j*-th voxel, and ${\rm{\Delta }}t$ being the time shift (see illustration in Fig. 2C). ${U}_{i}^{n}(D)$ being the tissue uptake of the *i*-th voxel at the *n*-th iteration step (Eq. 2).

We compared Eq. (1) parameters with diffusion coefficients measured using UV imaging in agarose hydrogels that is used for mimicking subcutaneous tissues^27,28^. Experimental results of insulin diffusion coefficients in different medium conditions ranges between 0.8–2.0 (m^2^/s). Similarly, the diffusion rates assigned for brain tissue in our simulations ranges between 0.4–2.1 (simulated volume/time). Specifically, an average of ~1.3 is seen at the striatum and the basal ganglia.

### Simulating tissue uptake as function of drug compounding

To calculate tissue uptake (i.e., the way the drug is compounded to increase tissue ability to absorb the drug), we define an absorption coefficient and saturation concentration threshold for each grid voxel (indicating the permissibility of the tissue at the overly area). We use the MRI intensity as indication of tissue density to derive the uptake parameters for the calculation. We assume correlation between tissue density and its absorption ability. Accordingly, we set higher uptake in grey areas (i.e., brain tissue) and lower uptake at black and white areas (liquid and bone, respectively). We approximate the absorption capacity as a specialized Gaussian function of MRI intensity where the maximum value is normalized to 1. The MRI intensity for maximum absorption is calculated as the Gaussian mean (*μ*) and the range of intensities, where significant absorption occurs is described by the Gaussian standard deviation (*σ*). The default solution with μ = 0.15 and *σ* = 0.04 is given in Fig. 2B. *μ* was derived from the distribution of MRI colors.

2 $${U}_{i}^{n}(D)={a}_{i}{D}_{i}^{n}$$

where *α*_*i*_ being the absorption coefficient of the *i*-th grid voxel (see illustration in Fig. 2C).

For comparison, experimental results calculated the glucose uptake value of 0.616 (g/min) in healthy brain tissue^28^. This result concurs with the range of 0.4–1.0 for tissue uptake in eBrain, specifically, an average simulated uptake of ~0.9 at the at the striatum and the basal ganglia. A more comprehensive comparison can be done against images taken using autoradiography approach with radiolabeled substances. The simulation can be fitted to the data, extract variables and calibrate the output for a more accurate results as well as to validate simulation predictions.

### Simulating tissue stimulation in the substantia nigra

Within the area of the substantia nigra we placed computational instances to indicate stimulation of the neurons within the virtual space. We marked the specific position of the tissue region on the MRI slice images to indicate where the neurons should be positioned. When the simulation is executed, it loads this information and identifies the grid voxel that overlap the marked region. At each run, the simulation places the instances randomly in the marked area, creating a uniform distribution of instances in a slightly different pattern. Instances carry a synthetic molecular stimulation mechanism that triggers a response once it senses a proper environmental signal. We assume that the stimulation is irreversible and there is no degradation of the intrinsic activity.

We tested the simulation with 1000 neurons (approximately four orders of magnitude less than typically in a 80 year old patient’s substantia nigra^29^) that are visualized as sphere-like globules that are colored green and are pulsing radially as an indication of internal metabolic activity. The amplitude of the scale change is adjusted according to its internal state. The cells remain green as long as they are not stimulated. Initial stimulation occurs once the neuron senses drug uptake of at least 0.33% of the maximal capacity of the voxel where it is positioned. Once a neuron triggers stimulation, it reflects the change by shifting its color to yellow, and then red upon reaching full stimulation. A fully stimulated cell terminates its pulse. Analysis of cell population scenarios was done using MATLAB R2017 by MathWorks (www.mathworks.com).

### Hardware requirements

The simulation was executed on an Intel Core i7-6700HQ Quad Core processor, running at 2.6 GHz under Windows 10 OS. Rendering was done using a Nvidia GeForce GTX 970M graphics card in a 1920 × 1080 resolution display. Under this setup we achieved a frame rate for the simulation of an average 7 ± 116 frames per second providing a smooth visualization of the simulated process.

## Section 2: eBrain Simulations of Drug Diffusion and Tissue Uptake

### Simulation of drug diffusion by intranasal drug delivery

We used the simulation to examine the dynamics of the intranasal delivery system though modification of the system parameters. We ran the simulation with a single theoretical drug that mimics diffusion of various possible subtracts. This drug encapsulates the key properties of a chemical substrate  (e.g., deferoxamine) or a protein (i.e., Insulin) that have diffusion ability through a tissue.

**Figure 3:**
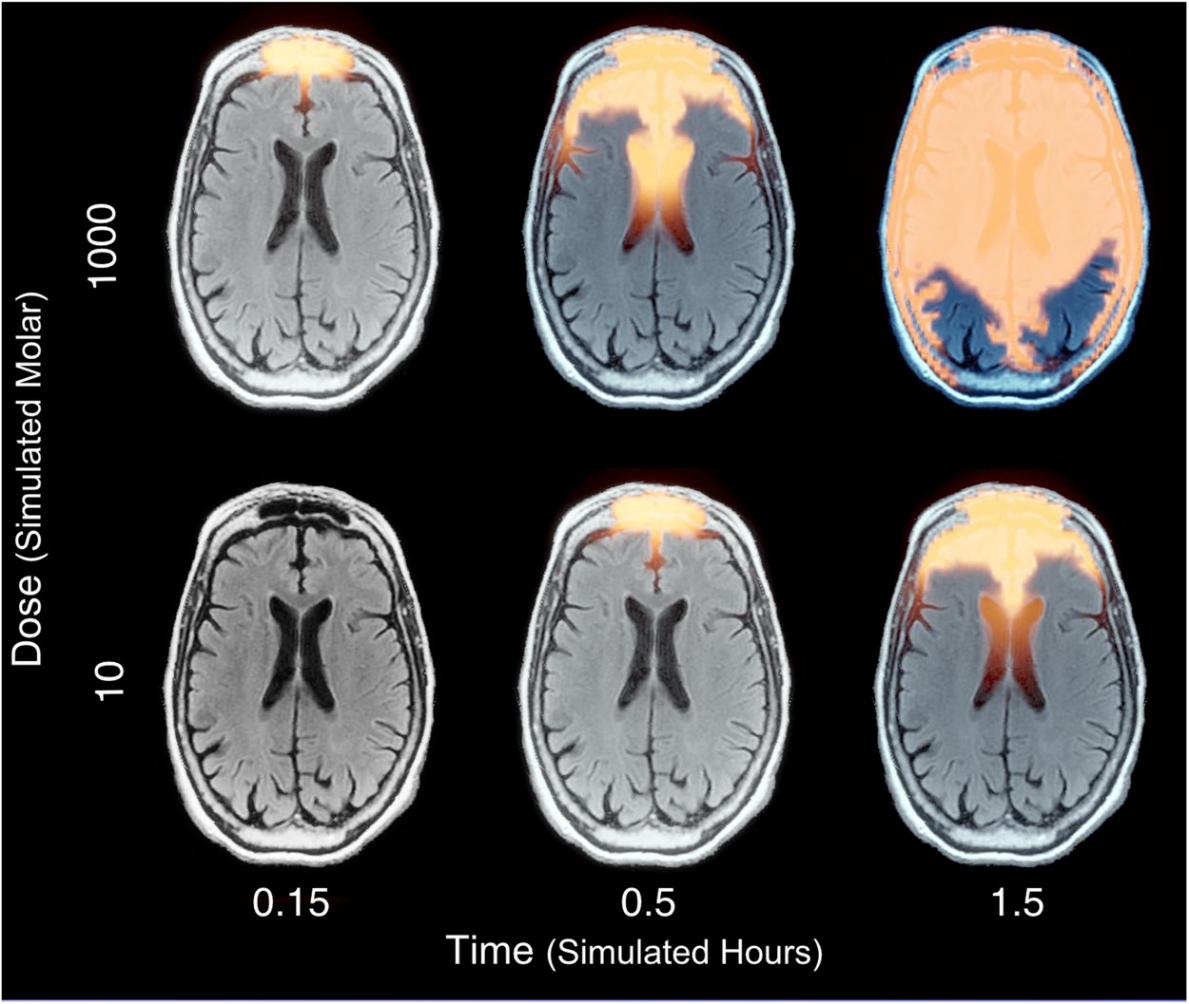
Drug diffusion in a slice of a brain MRI over the axial plane. Orange designates drug concentration. Sampling points at time 0.15, 0.5, 1.5 simulated hours under 10e^3^ and 1000e^3^ simulated Molar dose. See also Supplementary Clip 2.

To mimic intranasal infusion, we assigned simulated doses of the drug at the nasal passage regions of the MRI and studied its progression in the brain tissue (Fig. 3 and Supplementary Clip 2). Over time, the factor diffuses through the entire brain image over all slices and planes. Figure 3 displays snapshots of the diffusion at a specific brain slice over the axial plane under two doses, low (10e^3^ simulated Molar) and high (1000e^3^ simulated Molar) in three sampling points (0.15, 0.5, 1.5 simulated hours). The diffusion is not uniform across the environment, in the white areas, where the skull is positioned, the diffusion is blocked. In black areas, where fluids are present, the diffusion is faster. Whereas, in the grey areas where the brain tissue is found, the diffusion is decreased.

When the low dose is set in the simulation, at the first sampling point (~0.15 simulated hours) the drug did not diffuse through the axial plane to the slice indicated in Fig. 3. For the second sampling point (~0.5 simulated hours) the drug is predominantly localized in the most anterior regions of the brain but does not diffuse in the posterior direction. For the third sampling point (~1.5 hours), the drug diffuses posteriorly through half of the virtual slice, half way through the brain. When a high dose is infused, the diffusion occurs more rapidly. At the first sampling point the drug is already diffused through the axial plane to the indicated slice in Fig. 3 similar to the second sampling point in the low dose condition. In the second sampling time point for the high dose condition, the drug diffused posteriorly half way through the slice more similar to the third sampling point for the low dose condition. For the third sampling point in the high dose condition, the drug is fully diffused throughout the observed slice in Fig. 3. We observed an apparent shift in the diffusion dynamics between the two doses in which increasing the dose accelerated the diffusion between the sampling points.

**Figure 4:**
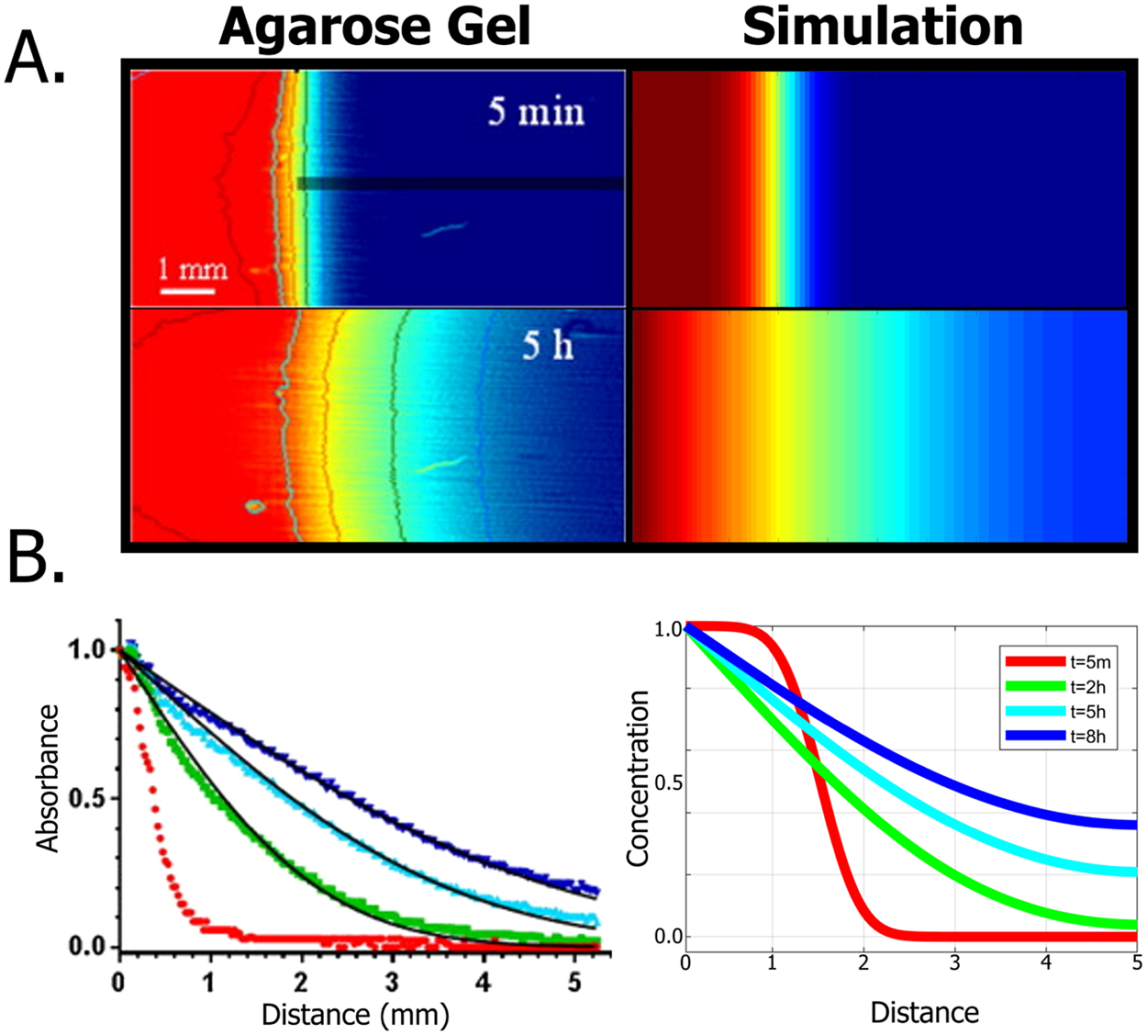
Insulin diffusion in agarose hydrogels compared with diffusion simulation (reproduced from 27; 1.0 sec corresponds to 1 iterations; 1.0 mm corresponds to 18 grid voxel (projected on a single axis)). (**A**) UV absorbance maps of insulin in agarose hydrogels (at 5.0 min and 5.0 hours; Left) versus similar maps of the diffusion simulations (Right). (**B**) Diffusion profiles of insulin in agarose hydrogels (Left) versus diffusion profiles as emerged in the simulation (Right).

To test the simulation against experimental data, we compared the diffusion in the brain tissue area with an experimental study in agarose hydrogels that are used for mimicking subcutaneous tissue^25^. We plotted the simulation and examined time-points that corresponded to the experimental tested scenarios. We observed a qualitative agreement between the simulation and the experimental results. Similar to the agarose gel assay (Fig. 4A), we see that the diffusion occurs gradually into the tissue. As time progresses, the substrate concentration spans over a larger space and the distribution degree drops. In early stages, the substrate is concentrated at the origin and a steep drop in concentration occurs as you move closer towards the target tissue, whereas at the later stage (5.0 hours) the concentration is gradually diffused within the tissue (Fig. 4B). Moreover, the concentration distribution curves concur between the simulation and the gel, showing an increasing rate over time (Fig. 4C,D). Due to the different nature of the experiments, the simulation shows a shift of the diffusion origin over time, particularly at the early stages (notice the red curve offset between the dynamics of experimental measurements and the simulation in Fig. 4C,D). Root mean square deviation (RMSD) of the results discloses an average error of 0.08 between the data and the simulation (see Supplementary Table S1 and Fig. S2).

### Simulation of tissue uptake of varying drug amounts

**Figure 5:**
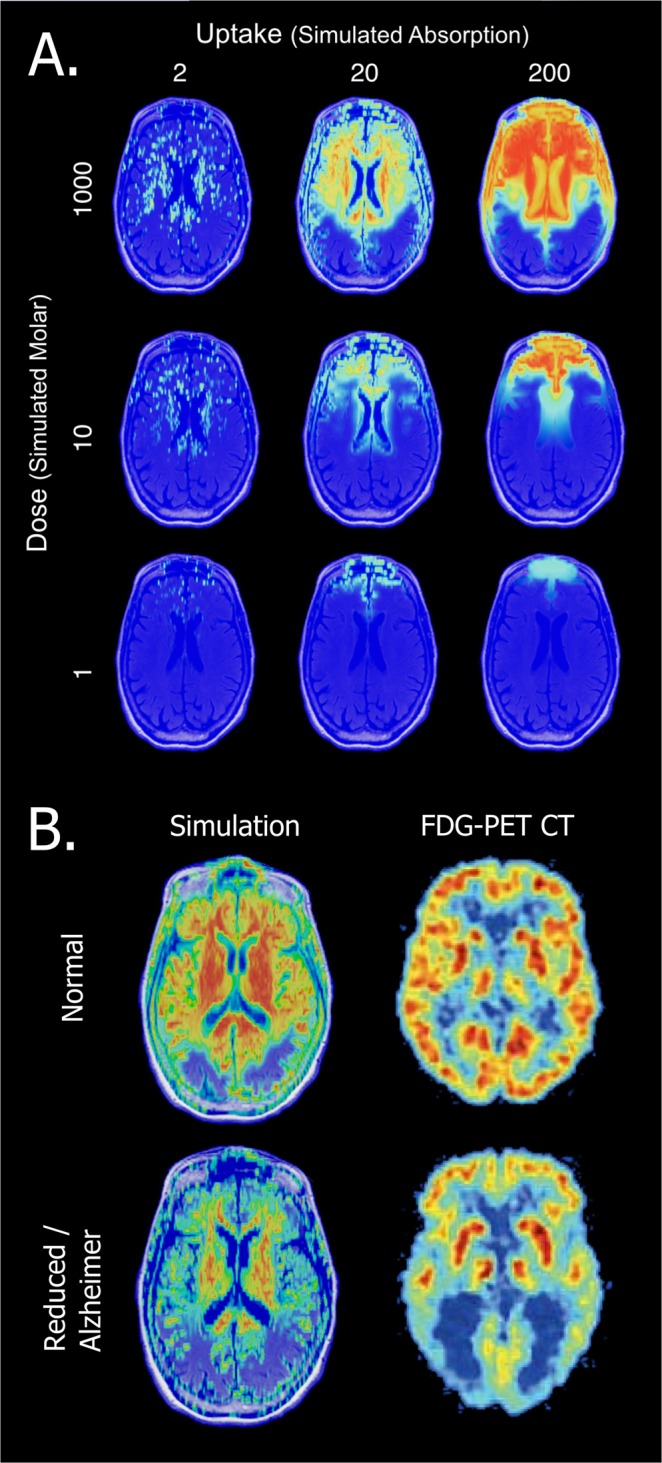
eBrain tissue uptake simulation. (**A**) Axial plane perspective under differed uptakes and doses parameters. Uptake levels are colored from low (blue) to high (red). Sampling points at time 1.0 simulated hour, dose: 1e^3^, 10e^3^ and 1000e^3^ simulated Molar and tissue uptake of 2e^−3^ 20e^−3^ and 200e^−3^ simulated absorption units. See also Supplementary Clip 3. (**B**) Comparison of the simulation with FDG-PET brain CT. Elderly adult whose glucose uptake is normal (Top) and Alzheimer’s disease patient whose glucose uptake is reduced (Bottom). The FDG-PET brain CT images in Subfigure B are not covered by the CC BY license. Image credit to Berti *et al*.^30^ and S. Karger AG, Basel. All rights reserved, used with permission.

We further used the simulation to study the tissue uptake of drug over time in the brain with different absorption abilities and drug doses. Figure 5A shows drug uptake by the tissue at a single sampling point (~1.0 simulated hour) under three different doses (rows; 1e^3^, 10e^3^, and 1000e^3^ simulated Molar) and three different tissue uptakes (columns; 2e^−3^, 20e^−3^ and 200e^−3^ simulated absorption units). Consistent with the diffusion results, increasing the dose makes enhances the tissue uptake. A low dose leads to tissue absorption in the most anterior regions of the brain, medium dose triggers absorption posteriorly halfway through the brain, and high dose promotes tissue uptake throughout the entire slice.

As expected, for low tissue uptake, we observed tissue responses scattered over the area where the drug is diffuse. When we increase tissue uptake to an intermediate level there is no response is observed in the fluid areas. Within the parenchyma there is an observed gradual absorption from the periphery to the middle of the brain. When we set extremely high uptake values, the tissue responds to its maximal magnitude. Moreover, in these extreme cases, even brain areas that are mostly occupied by fluids respond to the presence of the drug. This is possibly due to the minority of the cell population that is stirred in the fluid whose uptake is radically enhanced when absorption values get to an extreme state. Alternatively, this reflects the extremely enhanced absorptions of the tissues positioned outside of the observed axial slice (Fig. 5A).

To gain experimental support, we compared FDG-PET brain scan with slices of the MRI at approximately the same perspective plane (basal ganglia view of the axial plane). We tested two cases, an elderly adult whose glucose uptake is normal and an Alzheimer’s disease patient whose glucose uptake is significantly reduced in the brain^13,30^. The simulation, under baseline parameters, shows similar characteristics as the intensity of a normal subject FDG-PET scan, both show areas with higher uptake in the center of the tissue that gradually decreases at the peripheries and spans over most brain tissue (Fig. 5B, Top). In both cases, the ventricles show a low uptake activity (a blue colored in Fig. 5B, Top). We observed similarity in reduced activity simulation runs (uptake activity is threefold reduced) and the intensity of a FDG-PET scan of an Alzheimer disease subject (Fig. 5B, Bottom). In both cases, the activity amplitude is reduced showing only isolated islets in which the absorption reaches high magnitude, with a steep decrease in their surrounding areas. To evaluate the similarity between the simulation results and the FDG-PET scans (Fig. 5B, Bottom), we calculated the mean activity and the activity distribution. We found that the mean uptake value in the baseline simulation is 0.59 and drops to 0.4 in the reduced activity simulation. The mean uptake value was compared with the average density in the FDG-PET scans which was 0.52 and 0.41 for the normal subject and the Alzheimer’s disease subject, respectively (Supplementary Fig. S3 and Table S4). Additionally, we measured the activity distribution and calculated the areas of highest signal intensity in the overall brain tissue (value > 0.7 of the normalized data; Supplementary Fig. S3 and Table S4). We find that the baseline simulation data shows extensive uptake regions that occupy 22.8% of the brain tissue and the reduced activity simulations occupy 3.9% of the brain tissue. Similarly, high intensity areas occupy 19.3% and 5.6% of the brain tissue in the FDG-PET scans of the normal subject and the Alzheimer’s disease subject, respectively (Supplementary Fig. S5 and Table S4).

## Section 3: Study of Tissue Stimulation Over Time

### Tissue stimulation over time

**Figure 6:**
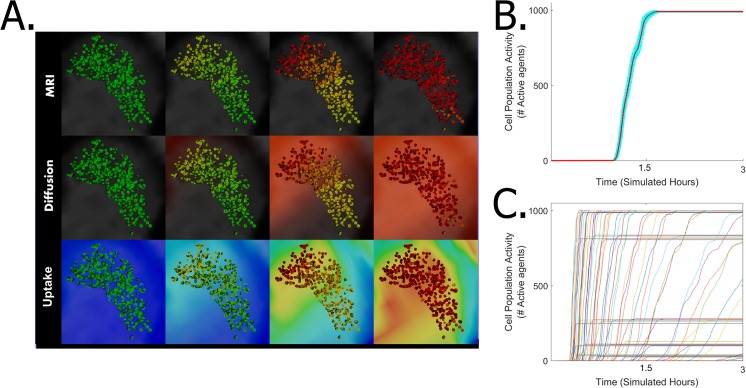
Stimulation of cell population at the Pars Compacta region of the substantia nigra (presented in three different modes, MRI (Top), diffusion (Middle) and tissue uptake (Bottom)). Four sampling points at time 0.33, 0.5, 0.66 and 0.833 simulated hours under tissue uptake of 40e^−3^ simulated absorption capacity units and dose of 100e^3^ simulate Molar. Cell in the native form are colored green, stimulated cells change their color to yellow, and to red when they are fully stimulated. See also Supplementary Clip 4. (**B**) Average of a repetitive 10 independent runs of the simulation with dose ~2e^3^ and uptake ~1e^−3^. Cyan indicates the error bar over the runs. (**C**) Multiple runs under various initial dose (1000e^3^:0.1e^3^ simulated Molar) and tissue uptake (100e^−3^:0.001e^−3^ simulated absorption capacity) conditions. Each curve represents a single run of the simulation.

We used the simulation to study how the drug stimulates the cell population at the substantia nigra over time. In general, the population is gradually stimulated once the factor diffuses to the substantia nigra location and absorbed by the tissue. As expected, as time progresses the population gradually changes the dominated default green color, into yellow color (indicating stimulation has started), until the entire population turns red (indicating that cells are fully activation). Figure 6 and Supplementary Clip 4 show the population at the Pars Compacta region of the substantia nigra at four sampling points (0,33, 0.5, 0.66 and 0.833 simulated hours), with specific dose (100e^3^ simulated Molar) and tissue uptake scenario (40e^3^ simulated absorption units). The samples are plotted in three different displays: the genuine brain MRI (Fig. 6, Top), over the diffused drug (Fig. 6, Middle) and over the simulated drug uptake into the tissue (Fig. 6, Bottom). Notably, there is an apparent positive correlation between the drug diffusion and the tissue uptake with the responsive state of the cells. To study the stimulation dynamics, we plotted the number of stimulated neurons over time (Fig. 6B; average of 10 independent simulation runs under dose of ~2e^3^ and uptake ~1e^−3^). We observed three distinct phases in the curve: (1) initial phase of the simulation, where the drug was not yet delivered to the substantia nigra region and thus the total number of stimulated cells equals zero. (2) activity phase, in which the drug was taken by the tissue, crosses the required threshold and the neuronal population is gradually stimulating its activity. (3) saturation phase, in which the population reaches equilibrium and maximal number of stimulated cells.

### Tissue stimulation core features

**Figure 7:**
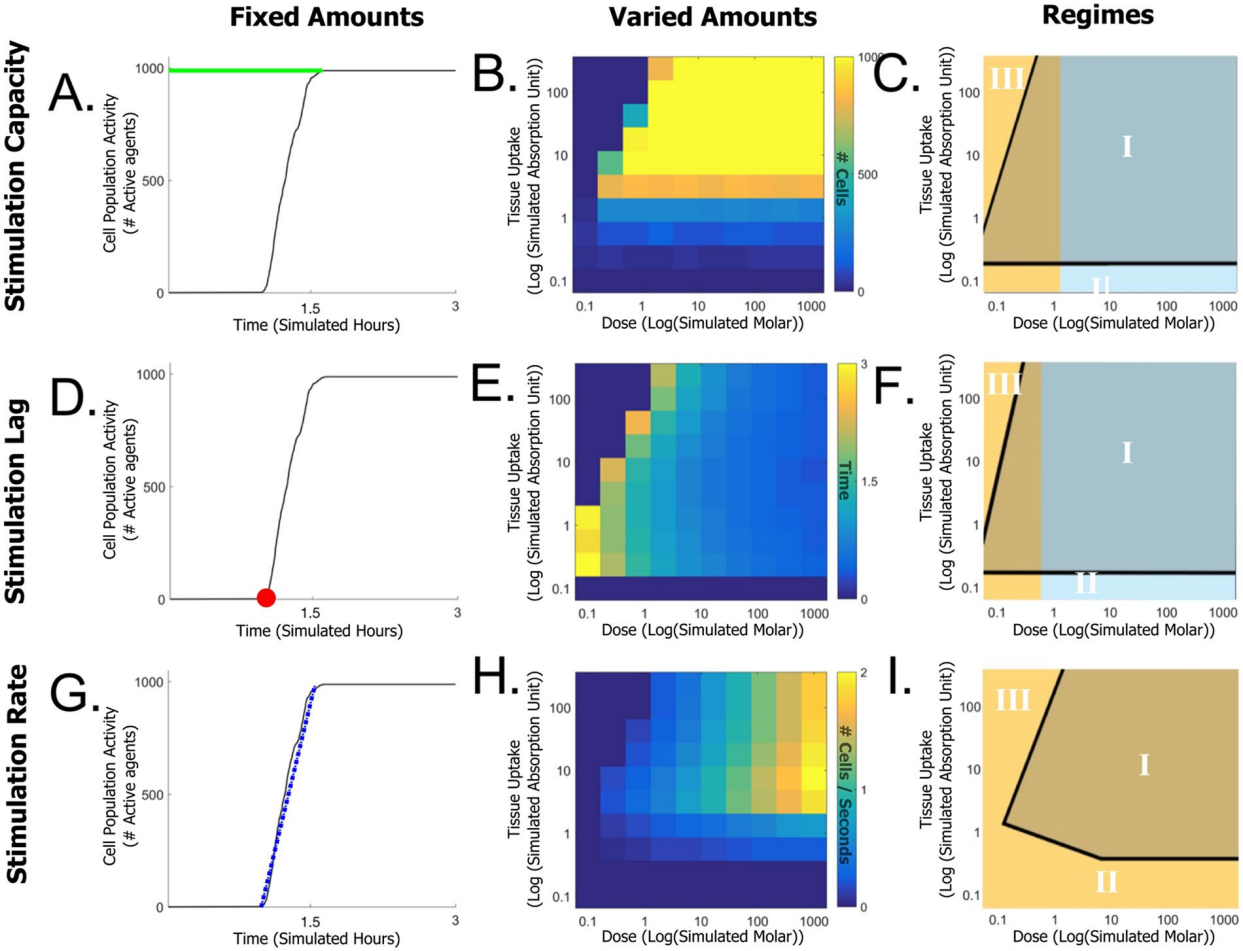
Internal functions of the simulation parameters extracted from multiple simulation runs. (**A**) Stimulation capacity (Green line) as calculated in a plot of neuron activity over time. (**B**) Capacity as function of dose and tissue uptake. (**C**) Schematic representation of capacity function regimes and activity domains. (**D**) Stimulation lag (Red star) as calculated in a plot of neuron activity over time. (**E**) Stimulation lag as function of tissue uptake and dose. (**F**) Schematic representation of stimulation lag function regimes and activity domains. (**G**) Stimulation rate (Blue line) as calculated in a plot of neuron activity over time. (**H**) Stimulation rate as function of tissue uptake and dose. (**I**) Schematic representation of stimulation rate function regimes and activity domain. The curve in left column is of a representative simulation run under dose ~2e^3^ and uptake ~1e^−3^. Maps in the middle column are calculated for 100 runs with different tissue uptake and dose (dose 100e^−3^:0.001e^−3^; uptake 100e^−3^:0.001e^−3^. Note the logarithmic scales). Schematic representation at the right column designate monotonic (Cyan) and skewed distribution (Orange) functions and the activity domains: I - activation area, II - no activity due to insufficient tissue uptake, III - no activity due to absorption at tissues prior to the substantia nigra). Notice the interdependency diagonal thresholds (Black).

To study the tissue stimulation process, we executed the simulation over one hundred different conditions (range of 1000e^3^-0.1e^3^ simulated Molar and 100e^−3^–0.001e^−3^ simulated absorption units). Each curve in Fig. 6C represents a single run of the simulation under specific dose and tissue uptake combinations. The simulation shows different curves for different combinations indicating that the initial dose and tissue uptake dominates the activity of the tissue. Specifically, we observed changes in the three key features of the dynamics of the stimulated neuron population: (1) The curves amplitude at the saturation phase varied between the runs, indicating variation in the stimulation capacity of the run. (2) The period of the initial phase before cells stimulation has been triggered, indicting varying stimulation lags. (3) The slope of the curve at the activity phase is different between the runs, indicating changes in the stimulation rates at the activity phase. These features can be defined by three core parameters: (1) stimulation capacity - the maximum count of simulated cells (i.e., reached its full activity potential at the saturation phase) (Fig. 7A), (2) stimulation lag - the period until tissue stimulation has been triggered (Fig. 7D) and (3) stimulation rate - the slope of tissue stimulation (calculated as the coefficient of a first order polynomial equation fitted to the curve (Fig. 7G).

The way in which these parameters behave as a function of the infused drug is not explicitly programmed and needs to be studied by an extensive analysis of the simulation under a vast range of input values. We plotted on a two-dimensional map for each parameter under multiple combinations of doses and tissue uptake (Fig. 7B,7E,7H). The maps display apparent distinct variations between simulations under different initial conditions. We verified that the dose and tissue uptake combinations elicit equilibrium for the functions we study (i.e., extending the analyzed range will have a minor effect on the results).

### Tissue stimulation as function of drug amounts

The map plots define a function in which each entry in the map indicates the amplitude of the response to a specific dose and tissue uptake combination (Fig. 7B,7E,7H). From this perspective, we observed that for a fixed tissue uptake (Y axis) value, the functions preserve the order as the dose concentration (X axis) increases: the stimulation capacity and rate show non-decreasing monotonic order (Fig. 7B,7H respectively) while stimulation lag shows non-increasing monotonic order (Fig. 7E). In contrast, for a specific dose concentration (X axis), the parameters as a function of the tissue uptake (Y axis) did not behave uniformly. Namely, stimulation capacity (Fig. 7B) and stimulation lag (Fig. 7E) act differently in two strict regimes: (1) non-decreasing monotonic regime where the function preserved an order and (2) skewed distribution regime where the function increases to a maximum point and then decreases (threshold between the regimes is ~5e^3^ and ~1e^3^ simulated Molar, respectively). Interestingly, the stimulation rate as function of tissue uptake (Fig. 7H) shows only a skewed distribution regime with no monotonic regime. The shape and amplitude of the skewed distribution function varied between the doses of each plot. For example, maximal stimulation rate of ~2.0 cells/sec is observed for dose of 1000e^3^ and simulated tissue uptake of 10e^−3^ simulated absorption units. The slope gradually decreases as tissue uptake increases (when reaches 150e^−3^ simulated absorption units, the stimulation rate drops to 1.5 cells/sec). A schematic description of the different regimes for each function for the tissue uptake values is given in Fig. 7C,7F,7I where cyan indicates a monotonic regime and orange indicates a skewed distribution regime.

While the monotonic regime is expected, the apparent skewed distribution regimes require further explanation. Skewed distribution prompts tissue uptake values that drive the optimal tissue stimulation. Below this value, drug absorption is reduced, whereas above this value, absorption is enhanced by the entire tissue and including in areas prior to the substantia nigra. Moreover, at an intermediate dose in the skewed distribution regimes, we observed that the response area is bound by an interdependency diagonal threshold. This analysis characterizes three activity domains: I - activation area, where the population is stimulated, II - no activity due to insufficient tissue uptake and III - no activity due to absorption at tissues prior to the substantia nigra (Fig. 7C,7F,7I). The interdependency diagonal thresholds (Black line) where higher doses compensate for lower uptake, and vice versa, in getting similar output (Fig. 7C,7F,7I). The stimulation capacity and stimulation lag show a single interdependency diagonal (approximately at 0.1e^3^–5e^3^ and 0.1e^3^–10e^3^ dose, respectively) whereas the activation rate shows two interdependency diagonals. One at the 0.1e^3^–1e^3^ dose range for low tissue uptake (0.1e^−3^–1e^−3^ simulated absorption units) and the other at the 0.1e^3^–10e^3^ dose range for high tissue uptake (1e^−3^–100e^−3^ simulated absorption units).

## Section 4: eBrain Analysis to Predict Optimal Treatment

**Figure 8:**
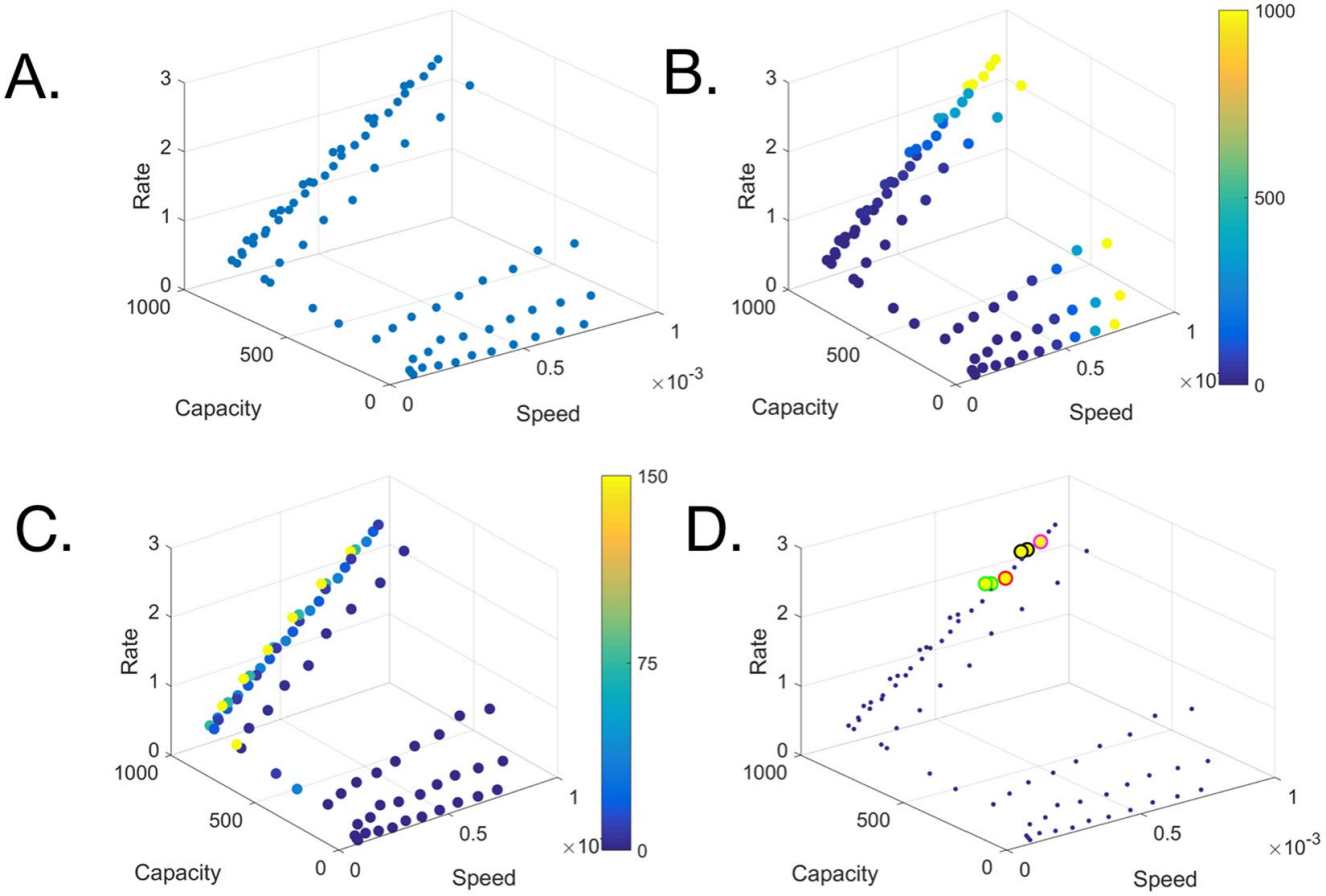
Screening optimal drug delivery over various possibilities. (**A**) 3D scatter plot of 100 treatment possibilities as function of their stimulation rate, speed and capacity (X, Y, Z axis, respectively, each data point defines a possible treatment). (**B**) Treatment possibilities labeled based on their dose quantity. (**C**) Treatment possibilities labeled based on their uptake level. (**D**) Optimal treatments screened from multiple possibilities (dose >30% and uptake >30% of their maximal value).

To predict the optimal treatment, we plotted dose and uptake combinations as function of the stimulation speed, rate and capacity (Fig. 8A). We observed two major clusters, one with high capacity (>700 stimulated cells) and one with low capacity (<400 stimulated cells). The minority of the treatment combinations are placed in the intermediate zone. These results correspond well with our previous analysis that revealed a steep incline from the base value to the maximum. To identify the optimal treatment, we colored the points to indicate the amplitude of the dose (Fig. 8B) and the uptake (Fig. 8C). As expected, the dose is directly correlated with the stimulation speed. Interestingly, the results show correlations of the treatment effectiveness with tissue uptake in which combining the treatment with an agent that maximally increases uptake does not necessarily lead to the maximal value symbolizing rate, speed, and capacity.

To identify optimal treatments that minimize resources while maximize impact, we highlighted the points where both values are above a specific threshold (30% of their maximal value). These points are highlighted in the space plot in Fig. 8D (uptake = 150, 73 and dose = 1000 (Black), uptake = 36 and dose = 360 (Red), uptake = 36 and dose = 1000 (Magenta) and uptake = 150, 73 and dose = 360 (Green)). Interestingly, the maximal points over the three functions were achieved by boosting to an immense dose while ignoring combination with other agents Nonetheless, the data point achieved by a combination of dose and addition of other agents are positioned within a relatively close vicinity.

Taken together, these results imply that efficacy of a drug can be increased by combining with additional agents at lower drug dose. As these are merely a fraction of the points, it may direct the decision on treatment based on specific individual needs. For example, if the subject cannot tolerate high doses or if there is a shortage of drug supply, the dose can be reduced and the tissue uptake increased by choosing higher permissibility compounds. Nonetheless, the treatment should be designed carefully to avoid over increasing tissue uptake that leads to consumption of the drug prior to the relevant area and consequently reducing the overall impact.

## Section 5: Future eBrain Extensions and Potential Applications

### Future extensions of eBrain

The eBrain prototype presented here can be considered as a proof of concept of a much broader vision of using 3D simulations in medical research. This study illustrates how eBrain reveals counter intuitive results, provides mechanistic explanations to observations and highlights underlying principles in intranasal drug delivery. Future renditions of eBrain will incorporate more molecular, physiological, and pathological data to help better predict disease progression and responsiveness to treatments.

We will focus eBrain to cover multiple delivery systems to allow better theoretical analysis of each delivery system. Future directions include comparing the systems effectiveness and classification of optimal delivery for treatments^31,32^. Along these lines, we have already implemented an intracerebral delivery system in which chemicals and cells are injected directly into the brain. Additionally, we are in the process of modeling the way molecules cross physical barriers, such as the Blood-Brain-Barrier (BBB) and the Brain Central Spline Fluid Barrier (BCSFB). Once implemented, eBrain will support intravenous, intragastric, and intrathecal delivery.

Furthermore, using various mathematical and computational techniques, we plan to extend eBrain to support physicochemical properties of potential drugs. We aim to build into our mathematical design elements to recapitulate form and stability of chemical formulas (e.g., molecular weight, drug half-life and clearance). We aim to incorporate more intracellular and intercellular mechanisms of the simulated neurons to support multiple molecular pathways and mechanisms. Each neuron would incorporate a program that mimics a neurons internal mechanisms^18,19^. This program will define regulation of receptors and neurotransmitters in molecular pathways (e.g., apoptosis, energy metabolism) and the function of cellular organelles (e.g., mitochondria, endoplasmic reticulum, and nucleus). To that end, we are on course to define a cellular program for dopaminergic neurons that spans from the substantia nigra to the striatum. We can then simulate neuronal response to oxidative stress by infusing toxins to study progression of cell death.

### eBrain applications in patient specific medical care

One of eBrain advantages is the ability to incorporate patient specific MRI data, from multiple time points and sources. Thus, eBrain can be used with time course MRIs to study the progressive effect of drugs in each sample. This enables monitoring of the treatment to predict changes in individuals. In the long run, eBrain can assist clinicians to prescribe the most relevant treatment, by simulating its impact on patient specific eBrain simulation together with known experience and population statistics. eBrain and similar attempts, will help develop more effective treatment paradigms to enhance personalized medicine beyond the ‘one-size-fits-all’ principle in which one protocol is administered to a large group of patients^8^. Development of this strategy will help clinicians in the future to adjust treatments to target populations of patients^33–35^.

The work discussed here illustrates how eBrain can contribute to the patient specific drug administration process as an additional layer to aid in treatment^16^. In addition, eBrain has the potential to impact other aspects in precision medicine. Clinical trial design can be improved through utilizing eBrain and executed in a more focused/refined manner. Similarly, eBrain can serve as an educational tool to train novices by illustrating brain activity. Moreover, eBrain can be used by the patients themselves to help understand their treatment, increasing their involvement in the medical process.

## Supplementary information

Supp Video 1

Supp Video 2

Supp Video 3

Supp Video 4

Supplementary information
