# Supplementary material for: eBrain: a Three Dimensional Simulation Tool to Study Drug Delivery in the Brain
Source: Sci Rep. 2019 Apr 16;9:6162. doi: 10.1038/s41598-019-42261-3 (PMC6467991; doi:10.1038/s41598-019-42261-3)
Supplement: Supplementary file 5 — Supplementary information [file 41598_2019_42261_MOESM5_ESM.pdf]

eBrain: a Three Dimensional Simulation Tool to Study Drug Delivery in the Brain

Yaki Setty*
Gateway Institute for Brain Research
*corresponding author: yaki.setty@gmail.com

Time point	RMSD
5 min	0.0717 (0.38)
2 hours	0.1138
5 hours	0.0669
8 hours	0.0682
Average	0.0801 (0.1572)

Supplementary Table S1: RMSD insulin diffusion in agarose hydrogels vs. diffusion simulation output at four different periods. RMSD was calculated over 100 sampling points (extracted from 1). Data at 5min was shifted 1.0mm to generate best fit. Raw data calculations is given in parentheses.

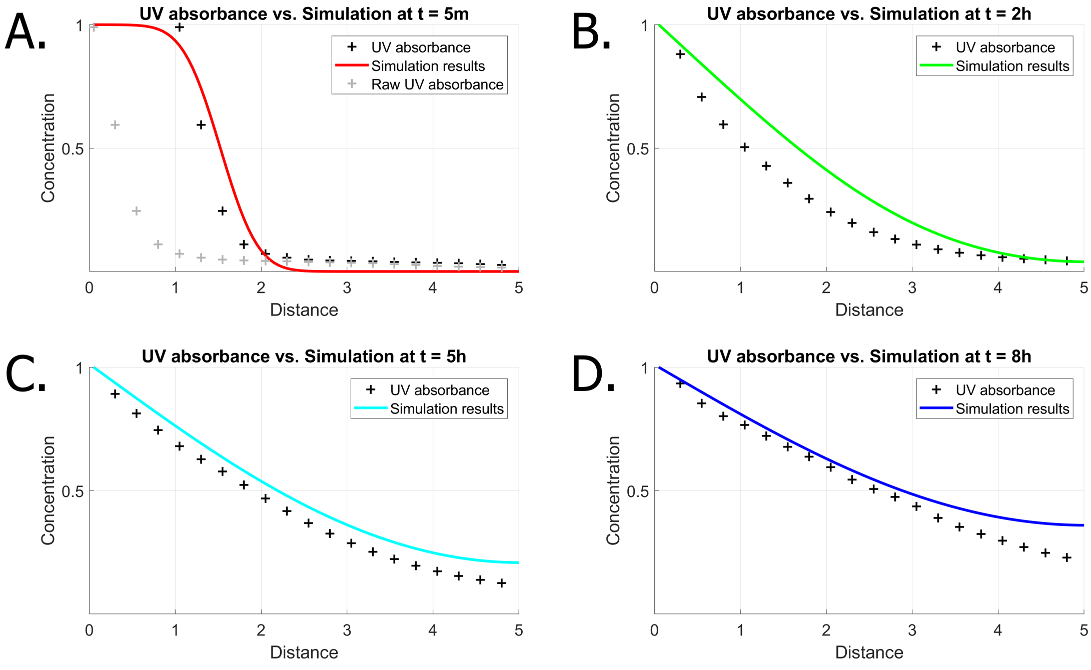

Supplementary Figure S2: Diffusion simulations compared with UV absorbance of insulin diffusion in agarose hydrogels (reproduced from 1; 1.0 sec corresponds to 1 iterations; 1.0 mm corresponds to 18 grid voxel (projected on a single axis)). A. Comparison at time point 5 min. Simulation data (red curve) vs. 25 points of raw data (grey plus sign) and raw data shifted 1mm (black plus sign). B. Comparison at time point 2 hours. Simulation data (green curve) vs. 25 points of raw data (black plus sign). C. Comparison at time point 5 hours. Simulation data (cyan curve) vs. 25 points of raw data (black plus sign). D. Comparison at time point 8 hours. Simulation data (blue curve) vs. 25 points of raw data (black plus sign).

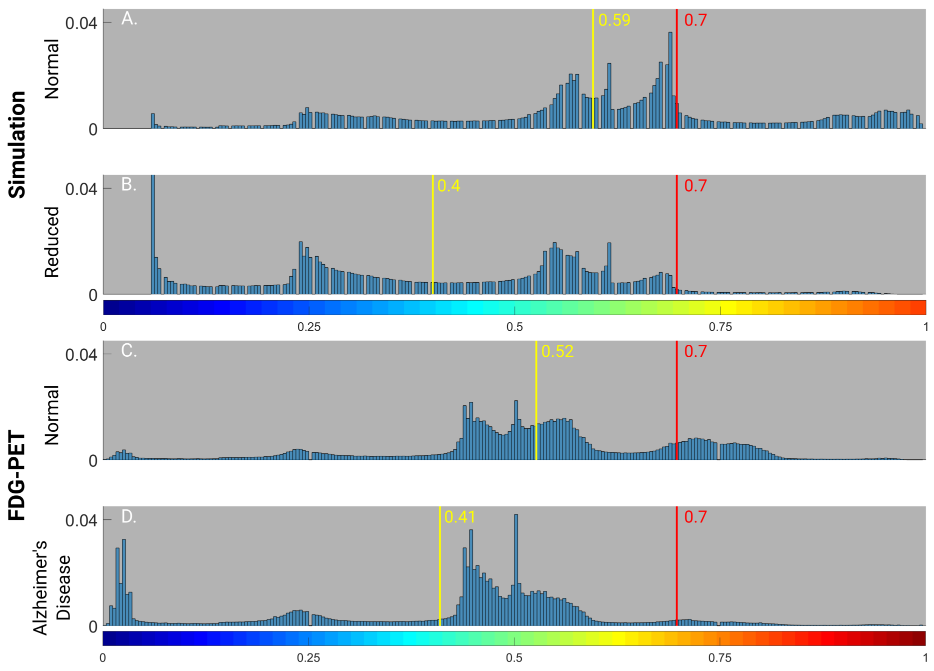

Supplementary Figure S3: Frequency of the uptake activity as function of activity level. Normalized distribution of uptake activity of snapshot from the simulation and FDG-PET scans (reproduced from 2). Values were normalized so that under baseline conditions and normal subjects to range of 0-1; the maximal value is set to 1 and the minimal value is set to zero. Red vertical line designates the threshold value (0.7); Yellow line designates the mean distribution activity. A. Simulation under baseline parameters. B. Simulation under reduced uptake activity. C. FDG-PET scan of a normal subject. D. FDG-PET scan of Alzheimer's disease subject.

	Extensive activity / overall tissue (%)	Mean activity (normalized units)
Simulation under baseline parameters	22.8	0.59
Simulation under reduced activity parameters	3.9	0.40
FDG-PET scan of a normal subject	19.3	0.52
FDG-PET scan of an Alzheimer's Disease subject	5.6	0.41

Supplementary Table S4: Tissue uptake analysis results. Extensive and mean uptake for the simulation results and FDG-PET scans for normal and reduced activity/Alzheimer's disease conditions. Extensive uptake activity/high intensity threshold was set to 0.7 of the maximal value under baseline parameters/normal subject data, respectively. Mean values were calculated for the uptake of the overall tissue region in normalized units of the values assigned to the brain tissue pixels (in the range of 0-1; 0 is no activity and 1 is maximal activity).

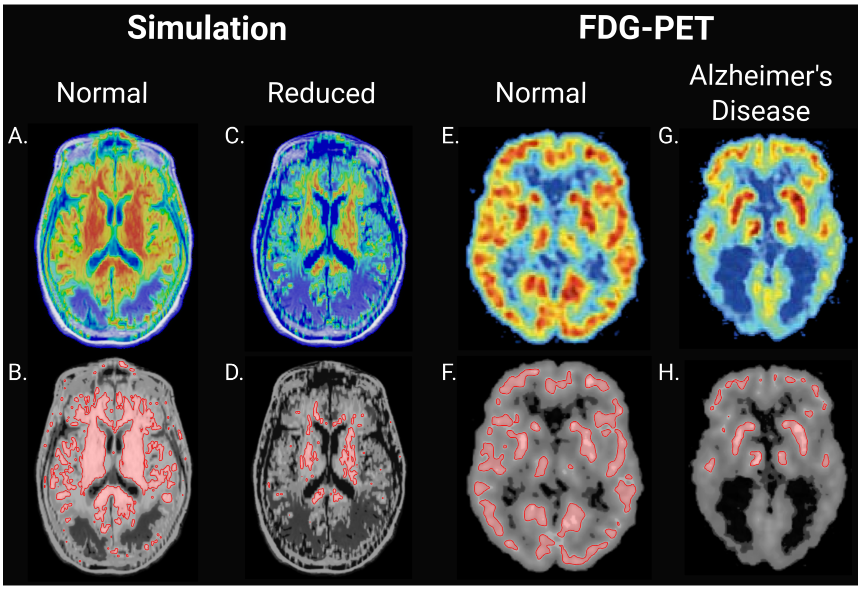

Supplementary Figure S5: Analysis of tissue uptake activity distribution in the simulation (snapshot taken at the axial plane at 1:39 hr) and FDG-PET scans (Reproduced from 2). A. Snapshot of the simulation under baseline parameters. B. Extensive activity regions (in red) contour superimposes the snapshot of the baseline simulation. C. Snapshot of the simulation of reduced uptake activity. D. Extensive activity regions (in red) superimposes the snapshot of the reduced activity simulation. E. FDG-PET scan of normal subject. F. high intensity regions (in red) superimposes the normal FDG-PET scan. G. FDG-PET scan of Alzheimer's Disease subject H. high intensity regions (in red) superimposes the Alzheimer's Disease FDG-PET scan. The images in Subfigure E and G are not covered by the CC BY license. Image credit to Berti et al. 2 and S. Karger AG, Basel. All rights reserved, used with permission.

Bibliography

1. Jensen, S. S., Jensen, H., Cornett, C., Moller, E. H. & Ostergaard, J. Insulin diffusion and self-association characterized by real-time UV imaging and Taylor dispersion analysis. *J Pharm Biomed Anal* **92**, 203-210, (2014).
2. Berti, V. *et al.* Early detection of Alzheimer's disease with PET imaging. *Neurodegener Dis* **7**, 131-135, (2010).
